# Transcranial ultrasound stimulation effect in the redundant and synergistic networks consistent across macaques

**DOI:** 10.1162/netn_a_00388

**Published:** 2024-12-10

**Authors:** Marilyn Gatica, Cyril Atkinson-Clement, Pedro A. M. Mediano, Mohammad Alkhawashki, James Ross, Jérôme Sallet, Marcus Kaiser

**Affiliations:** Precision Imaging, School of Medicine, University of Nottingham, Nottingham, United Kingdom; NPLab, Network Science Institute, Northeastern University London, London, United Kingdom; Department of Computing, Imperial College London, London, United Kingdom; Division of Psychology and Language Sciences, University College London, London, United Kingdom; Wellcome Centre for Integrative Neuroimaging (WIN), Department of Experimental Psychology, University of Oxford, Oxford, United Kingdom; Univ Lyon, Université Lyon 1, Inserm, Stem Cell and Brain Research Institute U1208, Bron, France; School of Computing Science, Newcastle University, Newcastle, United Kingdom; Rui Jin Hospital, Shanghai Jiao Tong University, Shanghai, China

**Keywords:** fMRI, High-order, TUS, Redundancy, Synergy

## Abstract

Low-intensity transcranial ultrasound stimulation (TUS) is a noninvasive technique that safely alters neural activity, reaching deep brain areas with good spatial accuracy. We investigated the effects of TUS in macaques using a recent metric, the synergy minus redundancy rank gradient, which quantifies different kinds of neural information processing. We analyzed this high-order quantity on the fMRI data after TUS in two targets: the supplementary motor area (SMA-TUS) and the frontal polar cortex (FPC-TUS). The TUS produced specific changes at the limbic network at FPC-TUS and the motor network at SMA-TUS and altered the sensorimotor, temporal, and frontal networks in both targets, mostly consistent across macaques. Moreover, there was a reduction in the structural and functional coupling after both stimulations. Finally, the TUS changed the intrinsic high-order network topology, decreasing the modular organization of the redundancy at SMA-TUS and increasing the synergistic integration at FPC-TUS.

## Author Summary

This article aims to elucidate how the transcranial ultrasound stimulation (TUS) could reorganize the brain as measured by the computation of redundancy and synergy. Our results showed that the TUS produced target-specific changes in the synergy minus redundancy rank gradient distribution at the limbic network at the frontal polar cortex (FPC-TUS) and the motor network at the supplementary motor area (SMA-TUS) and alterations in common, independent of the target, on the sensorimotor, temporal, and frontal networks. Moreover, the TUS changed the intrinsic high-order network topology, reducing the modular organization of the redundancy at SMA-TUS and increasing the synergistic integration at FPC-TUS.

## INTRODUCTION

Low-intensity transcranial ultrasound stimulation (TUS) is a promising and noninvasive neuromodulation technique that can safely alter neural activity and reach both cortical and deep areas with good spatial accuracy in comparison with other noninvasive brain stimulation methods (Bystritsky et al., 2011; Darmani et al., 2022; Legon et al., 2020). Although the exact mechanisms of TUS are still a matter of debate, some hypotheses have been suggested. At a microscopic level, TUS alters brain cells without causing a significant heating increase in the tissue (Naor, Krupa, & Shoham, 2016) through mechanical stimulation of sodium and calcium channels (Kubanek et al., 2016; Kubanek, Shukla, Das, Baccus, & Goodman, 2018; Tyler et al., 2008) and/or microcavitation resulting in local depolarization and alteration of the glia-neuron decoupling (Krasovitski, Frenkel, Shoham, & Kimmel, 2011; Oh et al., 2019; Plaksin, Kimmel, & Shoham, 2016). On a macroscopic level, TUS is related to an increased brain’s temperature without causing oedema or impairing the blood–brain barrier (Webb, Wilson, Odéen, & Kubanek, 2023) and to an increased excitability following a reduced GABA inhibition (Yaakub et al., 2023). TUS has also shown functional network alterations depending on the structural coupling of the target, with an increase in brain functional connectivity with the strongly connected areas and a decrease in correlations in the less-connected regions (Folloni et al., 2019; Verhagen et al., 2019) as well as behavioral changes (Fouragnan et al., 2019; Hameroff et al., 2013; Mahmoodi et al., 2024; Nakajima et al., 2022; Sanguinetti et al., 2020) from several minutes to several days following the stimulation.

However, studies are now required to understand how TUS could contribute to brain reorganization through high-order interdependencies explorations under the two types of interactions: redundancy and synergy. Redundancy can be understood as the repeated information we can obtain from any of the variables (*X*_1_ ∨ *X*_2_ ∨ … ∨ *X*_*N*_), where each of these variables represents a measure of brain activity for *N* brain regions (e.g., BOLD signals). Synergy is the extra information we get if we only observe the whole together (*X*_1_, *X*_2_, …, *X*_*N*_). Several mathematical and computational tools have been proposed to estimate them, whereas different studies have pointed out their relevance (Lizier, 2014; Rosas, Mediano, Gastpar, & Jensen, 2019; Timme, Alford, Flecker, & Beggs, 2014; Williams & Beer, 2010). A recent study reported redundancy linked with lower level sensorimotor processing and synergy with higher level cognitive tasks (Luppi et al., 2022), results that have also been replicated with a different decomposition framework of redundancy and synergy (Varley, Pope, Puxeddu, Faskowitz, & Sporns, 2023). Furthermore, they exhibited different network organization, with redundancy being more segregated and correlated with structural connectivity (SC). In contrast, synergy was associated with the wiring distance matrix between pairs of regions and favored integrated processing (Luppi et al., 2022). In healthy aging, redundancy increased in the older population (Camino-Pontes et al., 2018; Gatica, Cofré, et al., 2021) and could be explained by a nonlinear neurodegenerative model applied to the SC (Gatica, Rosas, et al., 2021). Furthermore, these high-order methods have been used in a wide range of studies, such as neurodegeneration (Herzog et al., 2022), artificial neural networks (Proca et al., 2024), spiking neurons (Stramaglia, Scagliarini, Daniels, & Marinazzo, 2021), and elementary cellular automata (Orio, Mediano, & Rosas, 2023; Rosas, Mediano, Ugarte, & Jensen, 2018).

This article aims to elucidate how the TUS could reorganize the brain as measured by the computation of redundancy and synergy at the individual level. In this direction, we reanalyze the TUS effect on resting-state fMRI data of three macaques under anesthesia, on a time period from 30 to 150 min following the stimulation (Verhagen et al., 2019). We independently characterized the high-order quantities at the control condition (non-TUS) and after applying TUS at two targets: the supplementary motor area (SMA-TUS) and the frontal polar cortex (FPC-TUS). Our results showed that the TUS produced target-specific changes in the rank gradient distribution at the limbic network at FPC-TUS and the motor network at SMA-TUS and alterations in common, independent of the target, on the sensorimotor, temporal, and frontal networks. Next, the differences were mostly consistent across macaques. Moreover, the TUS decreased the structural and functional coupling independent of the stimulated target. Finally, the TUS changed the intrinsic high-order network topology, reducing the modular organization of redundancy at SMA-TUS and increasing synergistic integration at FPC-TUS.

## RESULTS

**Figure 1.**
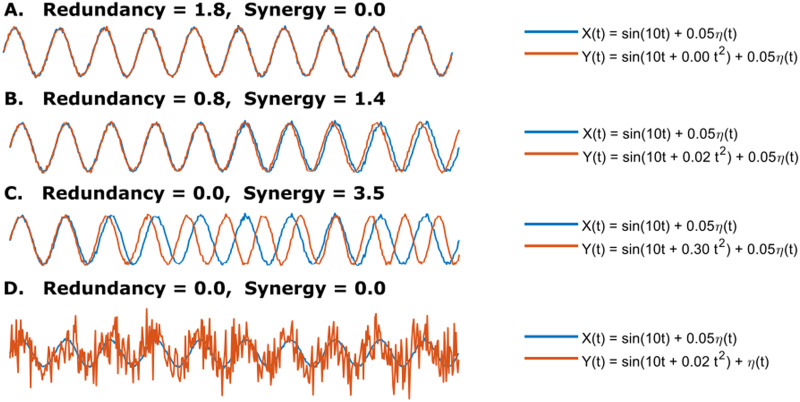
Given two random variables *X*(*t*) = *sin*(10*t*) + 0.05*η*(*t*) and *Y*(*t*) = *sin*(10*t* + *it*^2^) + 0.05*η*(*t*), with *i* = {0, 0.02, 0.3}, *t* ∈ [0, 2*π*], and *η* ∼ 𝒩(0, 1). (A) Full redundancy and zero synergy. (B) The redundancy decreases when *i* = 0.02. (C) Full synergy and zero redundancy when *i* = 0.3. (D) Synergy and redundancy are zero when *Y*(*t*) = *sin*(10*t* + 0.02*t*^2^) + *η*(*t*).

We first developed a simple example of two noisy sinusoids to give an interpretation of how the redundancy and synergy change when modifying one of the signals. The two random variables started highly correlated, resulting in high redundancy and synergy zero (Figure 1A), and after modifying the second one gradually, the redundancy decreased, and the synergy increased (Figure 1B) until the interaction is purely synergistic (Figure 1C). However, both quantities were annulled when the second random variable was noise-dominated (Figure 1D). Therefore, if we modulated one of the signals, we observed that the redundancy and synergy nulled at low and complete synchrony, respectively. In contrast, there was a region where both quantities coexisted.

Despite its lack of biological relevance, the inspiration from sinusoids aligns with works on coupled oscillators models (Cabral et al., 2014, 2022), where a zone of maximum metastability has been reported. Metastability consists of the brain transition between different configurations of synchronized states, quantified as the standard deviation of the Kuramoto order parameter (KOP), as a global variation between different states of synchrony (Orio et al., 2018; Tognoli & Kelso, 2014). Using a Hopf model (Coronel-Oliveros et al., 2024; Deco et al., 2019), we simulated BOLD-like signals with *N* = 140 oscillators coupled with the average SC (across the three macaques), reproducing regions where signals are globally synchronized over time (being fully synchronized when the mean of KOP is 1; Supporting Information Figure S1A), decreasing as noise is added (the extreme case, when mean of KOP is 0). Moreover, there is a zone with maximum metastability (Supporting Information Figure S1B; defined as the standard deviation of KOP) where neither redundancy nor synergy reaches their maximum values (Supporting Information Figure S1C).

**Figure 2.**
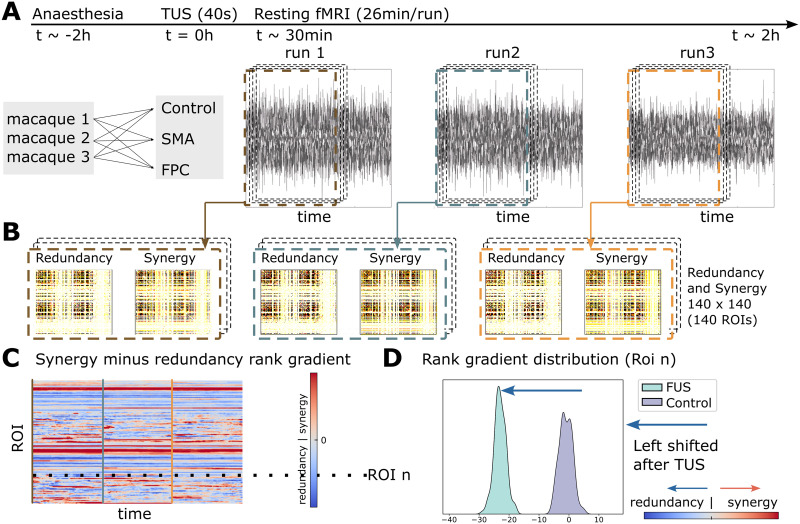
(A) Three macaques participated in FPC-TUS, SMA-TUS, and non-TUS. (B) We computed the redundancy and synergy matrices over 60 sliding windows of 500 s with 99% overlap. (C) The matrices are averaged across rows and then ranked. The result of those synergy minus redundancy vectors is the rank gradient (Luppi et al., 2022). (D) Per Region of interest (ROI), we compared the gradient rank distribution over time (dotted line) between the control and TUS, obtaining a shift to more redundant (blue arrow) or synergistic (red arrow) interactions.

Next, we analyzed fMRI data of three macaques at non-TUS, SMA-TUS, and FPC-TUS from 30 to 150 min following the stimulation (Figure 2A). To capture a region’s prevalence of redundancy or synergy, we performed a dynamical extension to the synergy minus redundancy rank gradient (Figure 2B–C). Redundancy and synergy were estimated using the integrated information decomposition (Φ*ID*) that decomposes the time-delay mutual information of two random variables (BOLD signals) into redundancy, synergy, and unique information (see the Materials and Methods section for details). The matrices are averaged across rows and then ranked. The result of that synergy minus redundancy vectors is the rank gradient (Luppi et al., 2022). Finally, to answer if some areas were shifted to more redundant or synergistic interactions after the stimulation, we compared the rank gradient distribution between each TUS experiment and the control condition per brain region (Figure 2D).

### Synergy-Redundancy Rank Gradient Disruption After SMA-TUS

**Figure 3.**
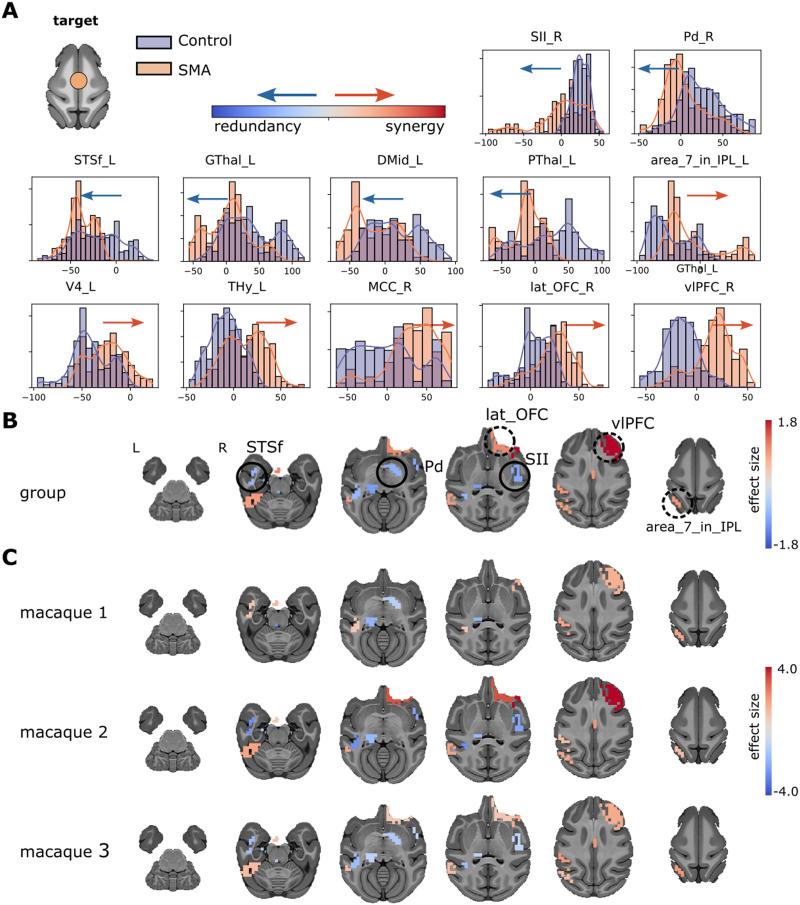
(A) Synergy minus redundancy rank gradient distribution after the TUS of the SMA target. The left shift (blue arrow) represents a region participating in more redundant (or less synergistic) interactions over time after TUS. In contrast, the right shift (red arrow) represents a more synergistic (or less redundant) interaction over time after TUS. (B) The brain maps illustrate the shift to the left (blue color) or right (red color), and the magnitude is the effect size. We compared the gradient rank distribution of each ROI over time between the control (non-TUS) and the TUS experiment among the three macaques together, using a Wilcoxon rank-sum test and correcting by Bonferroni and effect size bigger than 0.8. (C) Similar to B, but for each macaque separately.

To investigate the whole-brain effects of the stimulation at the SMA, we compared the synergy minus redundancy rank gradient between the non-TUS condition and the SMA-TUS. First, we grouped the three macaques and compared the gradient rank distribution between the control (non-TUS) and the SMA-TUS. Several networks were affected after the stimulation at SMA (Figure 3A). The sensorimotor area was altered in the right secondary somatosensory cortex (SII, es = −0.94) toward redundancy and the left inferior parietal lobule (area_7_in_IPL, es = 0.92) to synergy. The temporal area in the left fundus of the superior temporal sulcus (STSf, es = −0.80) participated in more redundant interactions. The frontal cortex, particularly the right lateral orbital frontal cortex (lat_OFC, es = 1.03) and the right ventrolateral prefrontal cortex (vlPFC, es = 1.80), shifted toward synergy. The motor area was altered to redundancy in the right pallidum (Pd, es = −0.87) and left posterior thalamus (PThal, es = −1.06). The effect size comparisons (SMA-TUS minus non-TUS) of those regions are shown on brain maps (Figure 3B). Moreover, at the individual level, the differences are homogeneous across two or three macaques (Figure 3C). Therefore, we found differences between non-TUS and SMA-TUS across the sensorimotor (↑ redundancy and ↑ synergy), frontal (↑ synergy), temporal (↑ redundancy), and motor (↑ redundancy) networks at group and individual levels.

### Synergy-Redundancy Rank Gradient Disruption After FPC-TUS

**Figure 4.**
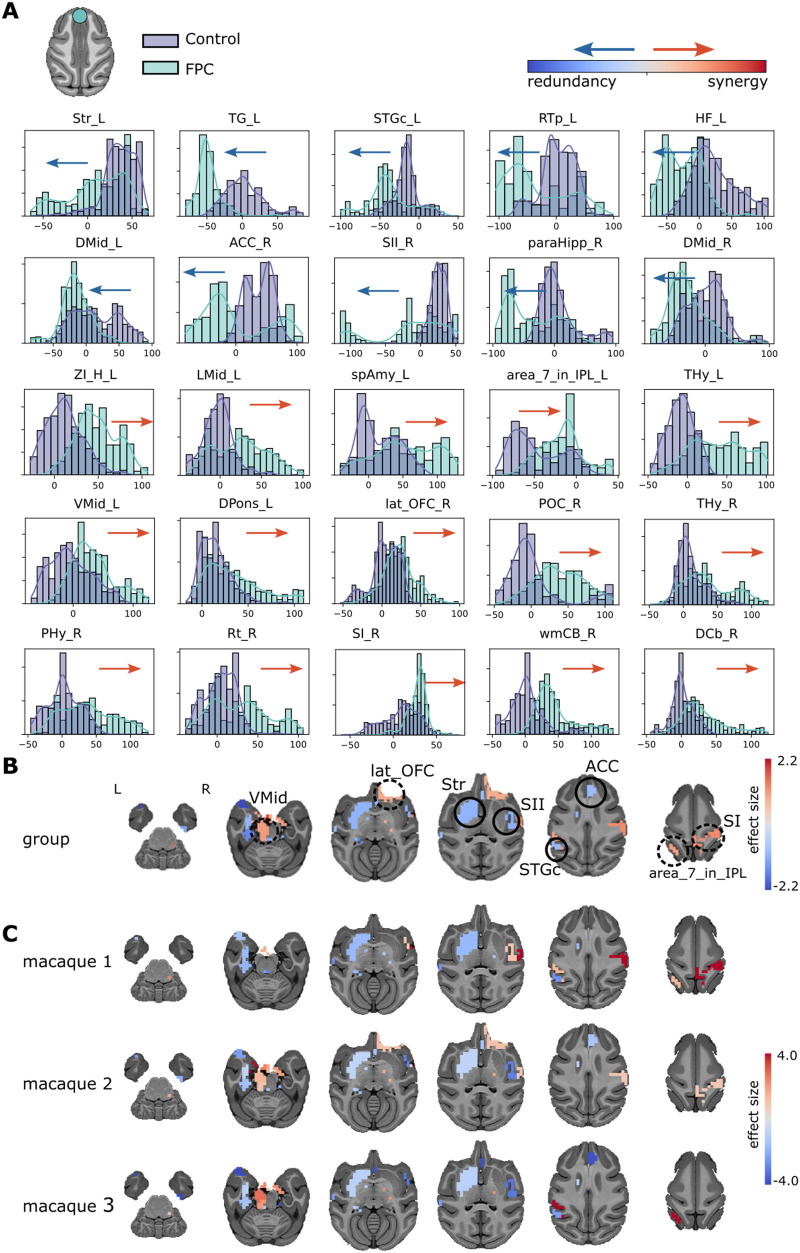
(A) Synergy minus redundancy rank gradient distribution after the TUS of the FPC target. The left shift (blue arrow) represents a region participating in more redundant (or less synergistic) interactions over time after TUS. In contrast, the right shift (red arrow) represents a more synergistic (or less redundant) interaction over time after TUS. (B) The brain maps illustrate the shift to the left (blue color) or right (red color), and the magnitude is the effect size. We compared the gradient rank distribution of each ROI over time between the control (non-TUS) and the TUS experiment among the three macaques together, using a Wilcoxon rank-sum test and correcting by Bonferroni and effect size bigger than 0.8. (C) Similar to B, but for each macaque separately.

To explore the network alterations of the stimulation at the FPC, we compared the synergy minus redundancy rank gradient between the control condition and the FPC-TUS. We found differences in the gradient rank distribution across several networks at the group level (Figure 4A). The sensorimotor network incremented the redundancy at the right secondary somatosensory cortex (SII, es = −1.07). In contrast, the sensorimotor region was altered toward synergy at the right primary somatosensory cortex (SI, es = 1.21) and the left inferior parietal lobule (area_7_in_IPL, es = 1.02). The temporal area shifted to redundancy at the left caudal superior temporal gyrus (STGc, es = −0.91). The frontal cortex switched toward synergy at the right lateral orbital frontal cortex (lat_OFC, es = 0.87). The limbic network shifted to redundancy at the left striatum (Str, es = −0.99), right hippocampus (paraHipp, es = −1.23), and right anterior cingulate cortex (ACC, es = −0.89). Additionally, the limbic network changed toward synergy at the left ventral midbrain (VMid, es = 1.03). The effect size comparisons of those regions are shown on brain maps (Figure 4B) for the group analysis. Most differences are consistent across two or three macaques, except the left amygdala, left pons, right lateral orbital frontal cortex, and right hypothalamus, which were statistically significantly different at only one macaque (Figure 4C). In conclusion, the stimulation at the FPC produced an effect on the somatosensory (↑ redundancy and ↑ synergy), temporal (↑ redundancy), frontal (↑ synergy), and limbic (↑ redundancy and ↑ synergy) networks.

### Structural and Functional Coupling

**Figure 5.**
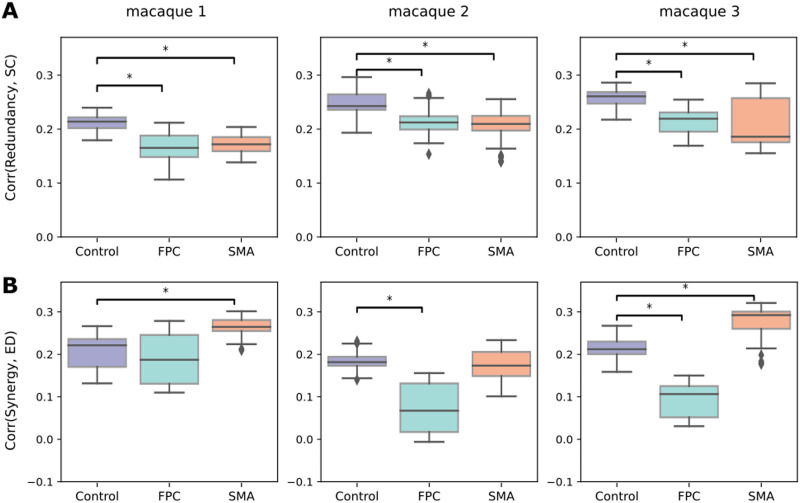
(A) Correlation between SC and redundancy. (B) Correlation between the ED and synergy per experiment and macaque (at each column). The *y*-axis values contain the Spearman’s rank correlation coefficient. The colors represent the control (non-TUS) and the two experiments: SMA-TUS and FPC-TUS. We corrected by Bonferroni and effect size bigger than 0.8.

To understand if there is an alteration in the functional and structural coupling produced by TUS, we quantified the similarities between the high-order quantities with the SC and the Euclidean distance (ED). The SC-redundancy correlation in controls was *ρ* = 0.24 and decreased to *ρ* = 0.198 at FPC-TUS. Moreover, it decayed to *ρ* = 0.196 at SMA-TUS, on group average (Figure 5A), and those differences persisted in all the macaques. Likewise, the ED-synergy correlation at non-TUS was *ρ* = 0.20 and decreased to *ρ* = 0.11 after FPC-TUS. Additionally, the differences were consistent in Macaques 2 and 3 (Figure 5B). Nevertheless, the similarity increased from *ρ* = 0.20 (non-TUS) to *ρ* = 0.24 at SMA-TUS, on average, across macaques. The consistent differences were observed in Macaques 1 and 3 (Figure 5B). Finally, the ED-synergy correlation was more significant than SC-synergy, and there was no apparent disruption in SC-synergy after TUS (Supporting Information Figure S2A). Similarly, the SC-redundancy correlation was more prominent than ED-redundancy and without significant disruptions at the ED-redundancy after any stimulation (Supporting Information Figure S2B). In conclusion, the SC-redundancy correlation decreased independent of the target. The ED-synergy similarity increased at SMA-TUS and decayed at FPC-TUS.

### Network Reorganization After TUS

**Figure 6.**
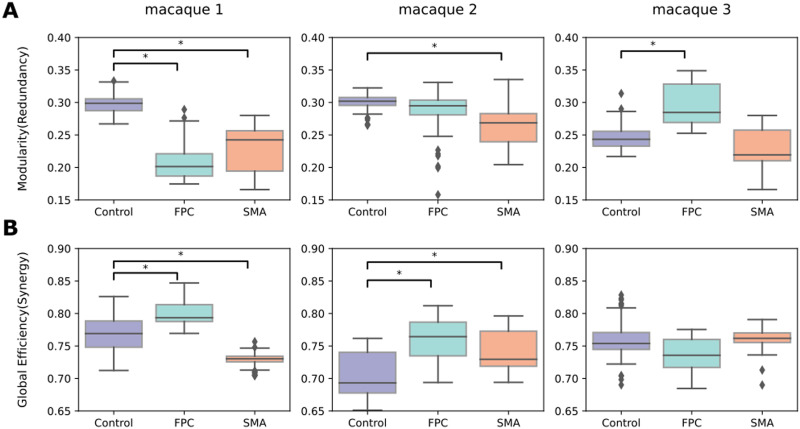
(A) Modularity (segregation) of the redundancy matrix. (B) Global efficiency (integration) of the synergy matrix. The colors represent the experiment or control condition, and each column depicts a macaque. We corrected by Bonferroni and effect size bigger than 0.8.

To further understand if a network reorganization exists in the functional high-order interactions, we seek to characterize the network topology of redundancy and synergy before and after the stimulation. We used modularity and global efficiency based on the pioneer topological characterization of redundancy and synergy. Redundancy exhibited a modular organization (Figure 6A), and synergy presented an integrated topology (Figure 6B). At the same time, the modularity in the synergy and the global efficiency in the redundancy were near zero in non-TUS or TUS experiments. Interestingly, the modularity of the redundancy decreased after the SMA stimulation (Figure 6A) in two macaques, with inconsistent changes in the synergistic global efficiency, presenting a decrease in the first macaque, an increase in the second, and no difference in the third (Figure 6B). In contrast, the FPC-TUS increased the synergistic integration (Figure 6B) without consistent alteration at the modular organization in the redundancy (Figure 6A). To compute modularity, we used the Newman algorithm for community detection that includes a resolution parameter, gamma. We reported the results for the default parameter *gamma* = 1. However, the cluster size would not be critical in altering the statistically significant modularity differences as long as there are at least two communities (Supporting Information Figure S3). While the absolute values of modularity change as the gamma parameter varies, the differences between TUS and control are qualitatively similar. Altogether, the intrinsic modularity in the redundancy decreased after SMA-TUS, and the integration in the synergy increased after FPC-TUS.

## DISCUSSION

**Figure 7.**
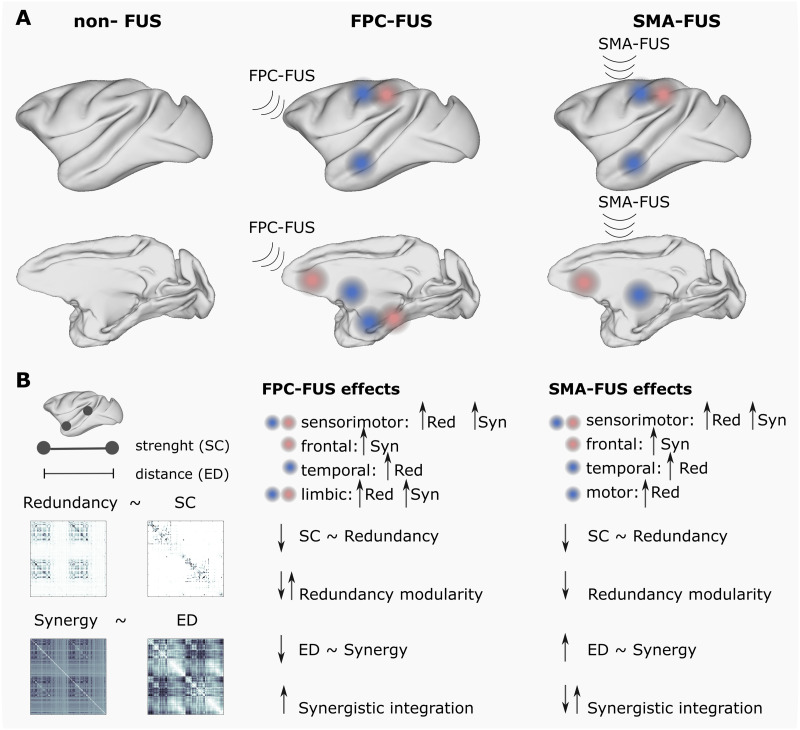
Overview. (A) The TUS produced target-specific changes in the rank gradient distribution at the limbic network at FPC-TUS and the motor network at SMA-TUS and alterations in common, independent of the target, on the sensorimotor, temporal, and frontal networks. (B) The TUS decreased the structural and functional coupling and altered, depending on the target, the intrinsic high-order network topology.

This article presented three main results: (a) The TUS produced high-order changes depending on the target at the limbic network at FPC-TUS and the motor network at SMA-TUS and altered, in both targets, the sensorimotor, frontal, and temporal networks (Figure 7A). (b) The differences were mostly consistent across macaques. (c) The TUS decreased the functional and structural coupling independent of the targets and modified the intrinsic high-order topological organization. The SMA-TUS decreased the modularity of the redundancy, and the FPC-TUS increased the synergistic integration (Figure 7B).

### SMA-TUS Changes the Rank Gradient Mostly Consistently Across Macaques

We found some regions changing to more redundant interactions at the sensorimotor (secondary somatosensory cortex), temporal (fundus of the superior temporal sulcus), and motor (pallidum and thalamus) networks. In contrast, some networks presented a shift to synergistic interactions, such as the sensorimotor (inferior parietal lobule) and frontal (ventrolateral prefrontal cortex and lateral orbital frontal cortex) networks (Figure 3). Although we used a high-order analysis instead of Pearson correlations, some regions are consistent with the previous study that analyzed the same data, reporting changes in the sensorimotor networks, prefrontal cortex, anterior temporal, and anterior and posterior cingulate at SMA-TUS on macaques (Verhagen et al., 2019). Moreover, targeting motor circuits on transcranial magnetic stimulation (TMS) produces changes not only in the somatosensory network (Bestmann, Baudewig, Siebner, Rothwell, & Frahm, 2004; Jung, Bungert, Bowtell, & Jackson, 2020) but also spread across different areas, for example, in the lateral frontotemporal cortex, including the inferior frontal gyrus (Pineda-Pardo et al., 2019), on human fMRI data.

### FPC-TUS Changes the Rank Gradient Mostly Consistently Across Macaques

There are some networks shifted to more redundant interactions at FPC-TUS, such as the sensorimotor (secondary somatosensory cortex), temporal (caudal superior temporal gyrus), and limbic (striatum, hippocampus, and anterior cingulate cortex). In contrast, other networks switched to more synergistic interdependencies, such as the sensorimotor (primary somatosensory cortex and the inferior parietal lobule), frontal (lateral orbital frontal cortex), and limbic (ventral midbrain; Figure 4). Even though we assessed a high-order analysis instead of Pearson correlations, several of these areas corresponded with the previous study that analyzed the same macaque fMRI data showing changes in the lateral prefrontal areas, the superior temporal sulcus, the posterior cingulate cortex, and the sensorimotor networks that presented functional connectivity differences after the FPC-TUS (Verhagen et al., 2019). On the other hand, the limbic network, particularly the striatum, thalamus, and amygdala, has modulated their functional connectivity after TMS at the frontopolar cortex in human data (Hanlon et al., 2013; Riedel et al., 2019). That network is also relevant for macaques, where the prefrontal cortex and the limbic network are widely connected (Carmichael & Price, 1995; Petrides & Pandya, 2007).

### TUS Decreases the Structure-Function Coupling

Redundancy showed a stronger correlation with SC, whereas synergy with distance, consistently with previous studies (Luppi et al., 2022). Those similarities were disrupted after TUS, with a global decrease in the correlations, except for the distance and synergy correlation that increased after SMA-TUS (Figure 5). The findings indicated that there may have been a high-order network reorganization driven by stimulation. At the level of pairwise correlations, previous studies have shown changes in the functional connectivity after TUS depending on the structural coupling of the target (Bestmann et al., 2004; Folloni et al., 2019; Pineda-Pardo et al., 2019; Verhagen et al., 2019), with an increase in the functional connectivity in the strongly connected areas, which are usually near the target.

### TUS Altered the High-Order Intrinsic Organization

Redundancy presented a modular organization, whereas synergy showed an integrated topology (Figure 6), consistent with previous studies (Luppi et al., 2022; Varley et al., 2023). A seminal study described integration and segregation as two underlying processes of brain organization that coexist, allowing to perform diverse cognitive tasks (Tononi, Sporns, & Edelman, 1994). Lower cognitive tasks have been linked with higher functional segregation in simple motor tasks. However, in working memory, tasks have been reported an increase of integration (Cohen & D’Esposito, 2016), where the prefrontal cortex had a critical role (Diamond, 2013; Menon & D’Esposito, 2022). In terms of high-order, lower-level processing, networks such as the motor area had been liked with a prevalence of redundancy, whereas the frontal cortex was predominated by synergy (Luppi et al., 2022). Interestingly, our findings also showed different high-order reorganizations depending on whether the target is the SMA or the prefrontal cortex, with the TUS altering the modular organization of the redundancy at SMA-TUS and, in contrast, increasing the intrinsic integration in the synergy at FPC-TUS.

### Limitations and Future Work

The current study has some limitations. First, rather than using absolute values, the synergy minus rank gradient depends on relative values dominated by redundancy and synergy. Second, we relied on the minimum mutual information (MMI) redundancy function, and there are other definitions that should be explored (Finn & Lizier, 2018; Ince, 2017). Nevertheless, our choice of this method is motivated by its simplicity in estimation and previous analysis pointing out the extent of these quantities. For instance, the loss of consciousness diminishes a synergy-based measure of integration in the human brain (Luppi et al., 2024). In artificial neural networks, redundancy has been linked to robustness, while synergy increases as the neural networks learn several tasks (Proca et al., 2024). The significance of redundant and synergistic interactions has been observed to support lower and higher level cognitive functions, respectively (Luppi et al., 2022). Notably, these findings have been recently corroborated using a distinct formalism of redundancy and synergy (Varley et al., 2023). Third, only three macaques were examined in this study, and larger sample sizes should be used in further research. Fourth, we analyzed macaque fMRI data under anesthesia. Previous research has demonstrated that each anesthesia treatment altered functional connectivity differently in the rat (Paasonen, Stenroos, Salo, Kiviniemi, & Gröhn, 2018), macaque (Giacometti et al., 2022), and human brain (Peltier et al., 2005). The dynamical functional connectivity has been investigated in macaques under anesthesia, visiting with higher frequency the functional connectivity patterns highly correlated with SC, as opposed to wakefulness where patterns with lower structure-functional coupling are the most explored (Barttfeld et al., 2015; Uhrig et al., 2018). In humans, similar findings have been replicated, showing a higher functional-structural coupling under anesthesia (Castro et al., 2024; Demertzi et al., 2019), and in high-order analysis, anesthesia reduces a synergy-based measure of integration (Luppi et al., 2024). Nevertheless, macaques were anesthetized using inhalational isoflurane gas based on a widely used protocol that preserves whole-brain functional connectivity (Neubert, Mars, Sallet, & Rushworth, 2015; Sallet et al., 2013; Vincent et al., 2007). Finally, resting-state fMRI data for human participants should be evaluated in future studies to move toward employing neuromodulation for therapeutic purposes.

### Conclusion

To the best of our knowledge, this is the first high-order analysis after TUS. Our results indicate that the redundant and synergistic interactions are altered after TUS with consistencies across macaques, and the patterns of changes depend on the target. Although using high-order interactions needs further research in TUS, they have been explored in many applications and might also be a relevant methodology in TUS experiments as a complementary approach to Pearson correlation.

## MATERIALS AND METHODS

### Ultrasound Stimulation

The ultrasound transducer device was a 64-mm diameter, H115-MR (Sonic Concepts, Bothell, WA, USA), with 51.74-mm focal depth, used with a coupling cone sealed with a latex membrane (Durex) and filled with degassed water. The protocol was controlled with a digital function generator (Handyscope HS5, TiePie engineering, Sneek, The Netherlands) setting to 250 kHz with 30-ms bursts of ultrasound generated every 100 ms. A 75-W amplifier (75A250A, Amplifier Research, Souderton) was used to deliver the power to the transducer. A TiePie probe (Handyscope HS5, TiePie engineering, Sneek, The Netherlands), connected to an oscilloscope, was used to monitor the voltage. The recorded peak-to-peak voltage remained constant throughout the stimulation. Per session, the voltages ranged from 130–142 V, analogous to 1.17–1.35 MPa, as measured in water with an in-house heterodyne interferometer (Constans, Deffieux, Pouget, Tanter, & Aubry, 2017). For the SMA target, the maximum peak pressure (P max) and I spatial-peak pulse-average intensity (Isspa) at the acoustic focus point estimations were 0.88 MPa and 24.1 W/cm^2^ (I spta: 7.2 W/cm^2^). For the FPC target, the parameters were 31.7 W/cm^2^ (I spta: 9.5 W/cm^2^) (Verhagen et al., 2019). The stimulation lasted for 40 s. The two target locations were close to the midline, stimulating both hemispheres simultaneously with a single train. The stimulation was guided using a frameless stereotaxic neuronavigation system (Rogue Research, Montreal), registering a T1-weighted MRI to each macaque’s head. The (*X*, *Y*, *Z*) Montreal Neurological Institute coordinates were (0.1, 2, 19) at the SMA and (−0.7, 24, 11) at the FPC. The positions of the transducer and the animal head were tracked continuously using infrared reflectors. The transducer was placed on previously shaved skin using conductive gel (Signagel Electrode, Parker Laboratories, Inc.) to ensure ultrasonic coupling between the transducer and the scalp. There were at least 10 days in between two TUS sessions. In the control condition (non-TUS), all procedures, including anesthesia, prescan preparation, fMRI scan acquisition, and timing, with the exception of the actual TUS, matched with the TUS sessions.

### Macaque Data Acquisition

The procedures were conducted under licenses from the United Kingdom Home Office following the Animals (Scientific Procedures) Act 1986. They all followed the European Union guidelines (EU Directive 2010/63/EU).

We used the offline TUS fMRI dataset of macaques from the repository (https://git.fmrib.ox.ac.uk/lverhagen/offlinetus). The data consists of three MRI sessions per control (non-TUS), SMA-TUS, and FPC-TUS, and macaque (*N* = 3). Each macaque was anesthetized using inhalational isoflurane gas (Neubert et al., 2015; Sallet et al., 2013; Vincent et al., 2007). Moreover, the macaques received injections of ketamine (10 mg/kg, intramuscularly), xylazine (0.125–0.25 mg/kg, intramuscularly), midazolam (0.1 mg/kg, intramuscularly), atropine (0.05 mg/kg, intramuscularlyly), meloxicam (0.2 mg/kg, intravenously), and ranitidine (0.05 mg/kg, intravenously). Following the stimulation, the animals were placed in a sphinx position in a 3 T MRI scanner (four-channel phased-array coil, Dr. H. Kolster, Windmiller Kolster Scientific, Fresno, USA). To avoid ketamine’s clinical peak, scanning started about 2 hours after anesthesia. Intermittent positive pressure ventilation was kept up to ensure a steady respiration rate. VitalMonitor software (Vetronic Services Ltd.) was used to record and monitor the values of respiration rate, inspired and expired CO_2_, and isoflurane concentration. Additionally, the core temperature and SpO_2_ were monitored constantly.

The fMRI data were acquired for three runs of 26 min each approximately using the scan parameters: 36 axial slices; in-plane resolution = 2 × 2 mm, slice thickness = 2 mm, without slice gap, TR = 2,000 ms, TE = 19 ms, and 800 volumes per run. A T1-weighted structural MRI was scanned per macaque. Image acquisition was performed following a T1-weighted magnetization-prepared rapid-acquisition gradient echo sequence with voxel resolution = 0.5 × 0.5 × 0.5 mm.

Finally, a T1-weighted structural MRI scan was acquired using a T1-weighted magnetization-prepared rapid-acquisition gradient echo sequence (voxel resolution: 0.5 × 0.5 × 0.5 mm), a black bone (voxel resolution: 0.5 × 0.5 × 0.5 mm), and a diffusion-weighted imaging (DWI) (voxel resolution: 1 × 1 × 1 mm) scans, per macaque.

### fMRI Preprocessing

The fMRI preprocessing was performed following previous pipelines using AFNI (Cox, 1996; Cox & Hyde, 1997): First, the T1 structural image was realigned to match the resting state. Next, the skull of the T1 image was removed. The cerebrospinal fluid (CSF) and gray and white matter were segmented and realigned to the template space. Next, a slice-time correction, despike, and motion correction were applied to the fMRI data; each volume was aligned with the mean volume; and a motion correction was applied. Then, the preprocessed fMRI data were spatially normalized to the NMT v2.0 brain template, and the CHARM (Jung et al., 2021; Reveley et al., 2017) and SARM (Hartig et al., 2021) atlases (level 3) were applied. Next, we detrended the fMRI using motion as a nuisance variable. Therefore, the original time series were grouped into 140 brain regions, and finally, a band-pass filter between 0.0025 and 0.05 Hz was applied (Barttfeld et al., 2015).

### Diffusion Preprocessing

The diffusion data were preprocessed using MRtrix. The pipeline consisted of denoising, Gibbs-ringing artifact removal, Eddy current, and bias field correction. Next, probabilistic tractography was performed using multishell, multitissue constrained spherical deconvolution (CSD). First, 10 million streamlines were generated with the gray matter-white matter interface optimization. Then, we applied a spherical-deconvolution-informed filtering of tractograms and reduced the number of streamlines to 1 million. Next, the connectome was computed on the CHARM (Jung et al., 2021; Reveley et al., 2017) and SARM (Hartig et al., 2021) atlases (level 3), resulting in a 140 × 140 SC matrix, per macaque, by counting the number of white matter streamlines connecting all module pairs.

### Synergy Minus Redundancy Rank Gradient

#### Partial Information Decomposition (PID).

Given three random variables, two source variables *X*^*i*^ and *X*^*j*^, and a target *Y*, the PID (Williams & Beer, 2010) is given by:

$$Red\left(X^{i},X^{j};Y\right)+Syn\left(X^{i},X^{j};Y\right)+\text{Unique}\left(X^{i};Y\right)+\text{Unique}\left(X^{j};Y\right),$$

in which *Red*(*X*^*i*^, *X*^*j*^; *Y*) is the information provided by *X*^*i*^ and *X*^*j*^ about *Y* (redundancy), *Syn*(*X*^*i*^, *X*^*j*^; *Y*) is the information provided by *X*^*i*^ and *X*^*j*^ together about *Y* (synergy), *Unique*(*X*^*i*^; *Y*) is the information that is provided only by *X*^*i*^ about *Y*, and *Unique*(*X*^*j*^; *Y*) is the information that is provided only by *X*^*j*^ about *Y*. The PID could be represented by a forward decomposition into the nodes: 𝒜 = {{12}, {1}, {2}, {1}{2}}, being the nodes the synergistic, unique in source 1, unique in source 2, and redundant information, respectively.

We followed the MMI PID decomposition on Gaussian systems, where redundancy is computed as the minimum information between each source and the target, and synergy refers to the additional information provided by the weaker source when the stronger source is known (Barrett, 2015).

#### Φ*ID*.

Consider the stochastic process of two random variables *X*_*t*_ = {$X_{t}^{i}$, $X_{t}^{j}$} and denote the two variables in a current state *t*, by $X_{t}^{i}$ and $X_{t}^{j}$, and the same two variables in a past state *t* − *τ*, by $X_{t-\tau }^{i}$ and $X_{t-\tau }^{j}$. The Φ*ID* is the forward and backward decomposition of *I*($X_{t-\tau }^{i}$, $X_{t-\tau }^{i}$; $X_{t}^{i}$, $X_{t}^{i}$), called the time-delay mutual information, in redundant, synergistic, and unique information (Mediano et al., 2021). Therefore, the Φ*ID* could be represented with the forward and backward interactions of the product 𝒜*x*𝒜. It results in 16 atoms (synergy to synergy, redundancy to redundancy, unique in source 1 to unique in source 2 (and backward), and redundancy to synergy, to name a few)). In this article, we are focused on two atoms: persistent redundancy (redundancy that continues being redundancy) and persistent synergy (synergy that continues being synergy).

#### Synergy minus redundancy rank gradient.

We performed a dynamical extension to the synergy minus redundancy rank gradient (Luppi et al., 2022). First, we computed the redundancy and synergy matrices measured with Φ*ID* over each sliding window. The Φ*ID* was computed over all the pairwise BOLD signal combinations {$X_{t}^{i}$, $X_{t}^{j}$}, *i* and *j* being two different brain regions, with (*i*, *j*) ∈ {1, …, 140}, with the time series truncated to each sliding window. Next, each redundancy and synergy matrix belonging to the same window was averaged across rows separately, obtaining two strength vectors (each 1 × 140) and ranking their participation based on their strength. The rank gradient consists of the synergy minus redundancy rank, obtaining one vector (1 × 140) per window. In particular, each run lasted around 26 min, with 800 volumes or time points. We defined 500-time points per window with 99% overlap, resulting in 60 windows. As every session (TUS or non-TUS) and each macaque included three runs, we concatenated the synergy minus redundancy rank gradient of all the 3 × 60 = 180 windows, resulting in a matrix (140 × 180) per macaque and experiment. We compared the three macaque sessions concatenated (dim 140 × 540 each gradient rank matrix) for global analysis at non-TUS versus each TUS experiment. In contrast, for the case of individual comparisons, we used only the gradient rank of each macaque control versus the TUS experiment (dim 140 × 180 each).

### Similarity

This analysis followed previous findings, reporting the different structural support for the high-order quantities, where redundancy was correlated with the SC and synergy with the distance (Luppi et al., 2022). Because the SC is a sparse matrix, the structure-function correlations were computed over the connected nodes. Therefore, the redundancy and synergy matrices were thresholded over the nonzero weight of the SC. Then, Spearman’s rank correlation coefficient was assessed between the upper triangular part of the thresholded SC and the upper triangular part of the redundancy (or synergy) matrix. In contrast, no thresholds were applied when comparing the distance and high-order correlations. We computed Spearman’s rank correlation coefficient between the upper triangular parts of the ED matrix and redundancy (or synergy).

### Network Analysis

We performed the graph analysis for weighted networks using the Brain Connectivity Toolbox (BCT) implementations (Rubinov & Sporns, 2010) over the redundancy and synergy matrices that, by definition, have nonnegative values. We used the BCT’s Python function bct.efficiency_wei to calculate the global efficiency. The input’s function is *W*, representing the redundancy or synergy matrices. Then, the weights are inverted using an auxiliary connection-length matrix *L*, *L*_*i*_*j* = 1/*W*_*i*_*j*. Finally, the global efficiency is computed over the matrix *L*. We used the Python function bct.community_louvain to compute segregation. The modularity detection algorithm includes one free parameter, gamma, which controls the resolution of the clusters. Larger clusters are detected when gamma is between 0 and 1, while values greater than 1 result in smaller clusters. The default parameter used in this manuscript was gamma = 1.

#### Segregation.

To quantify segregation, we used *modularity*, which enables the subdivision of the network into nonoverlapping modules densely interconnected within each cluster and weakly connected between modules. The *modularity* (*Q*) was estimated using Newman’s spectral community detection algorithm (Newman, 2006; Reichardt & Bornholdt, 2006). Mathematically, for a weighted graph, it is defined as:

$$Q=\frac{1}{2m}\sum_{i,j\in N}\left[a_{ij}-\frac{k_{i}k_{j}}{2m}\right]\delta \left(m_{i},m_{j}\right),$$

where *k*_*i*_ is the degree of the node *i*, *m*_*i*_ is the community of the node *i*, *m* is the sum of all of the edges in the graph, and *δ* is the Kronecker delta function (*δ*(*x*, *y*) = 1 if *x* = *y*, 0 otherwise).

#### Integration.

To characterize the integration, we quantified the *global efficiency*, which is the inverse of the average shortest path length connecting two regions (Latora & Marchiori, 2001), meaning that, for disconnected nodes, their efficiency is zero. The *global efficiency* (*E*) is defined as:

$$E=\frac{1}{n}\sum_{i\in N}E_{i}=\frac{1}{n}\sum_{i\in N}\frac{\sum_{j\in N,j\neq i}d_{ij}^{-1}}{n-1},$$

with *E*_*i*_ as the efficiency of node *i* and *d*_*ij*_ as the shortest path connecting the node *i* with the node *j*.

### Statistical Analyses

This study compared the control (non-TUS) with each TUS experiment (FPC-TUS or SMA-TUS). First, we performed a global analysis between the control and each TUS experiment, grouping the three macaques together and then at the individual level. The nonparametric statistical Wilcoxon rank-sum test assessed the group and individual differences. We used Bonferroni correction and considered only the differences with an effect size bigger than 0.8. Finally, for the macaque-level analysis, besides Bonferroni and effect size correction, we constrain the areas showing differences to the regions belonging to the group mask of differences.

### Code Availability

The data analysis was performed in MATLAB version 2022b. The MATLAB code to quantify synergy and redundancy from Φ*ID* of time series with the Gaussian MMI solver is available at https://doi.org/10.1038/s41593-022-01070-0 (Luppi et al., 2022). The MATLAB code to assess the network analysis is freely available at http://www.brain-connectivity-toolbox.net/ (Rubinov & Sporns, 2010). We downloaded the 3D macaque brain template from the Scalable Brain Atlas web page (https://scalablebrainatlas.incf.org/; Bakker, Tiesinga, & Kötter, 2015; Markov et al., 2014).

## ACKNOWLEDGMENTS

M.K., J.R., and C.A. were supported by the Engineering and Physical Sciences Research Council (EP/W004488/1 and EP/X01925X/1). M.K. was also supported by the Guangci Professorship Program of Rui Jin Hospital (Shanghai Jiao Tong University). J.S. is funded by a Sir Henry Dale Wellcome Trust Fellowship 105651/Z/14/Z, IDEXLYON IMPULSION 2020 grant (IDEX/IMP/2020/14) and French National Research Agency grant (ANR-22-CE37–0021). The Wellcome Centre for Integrative Neuroimaging is supported Wellcome Trust core funding 203139/Z/16/Z.

## SUPPORTING INFORMATION

Supporting information for this article is available at https://doi.org/10.1162/netn_a_00388.

## AUTHOR CONTRIBUTIONS

Marilyn Gatica: Conceptualization; Data curation; Formal analysis; Investigation; Methodology; Visualization; Writing – original draft; Writing – review & editing. Cyril Atkinson-Clement: Conceptualization; Data curation; Investigation; Methodology; Writing – original draft; Writing – review & editing. Pedro A. M. Mediano: Conceptualization; Investigation; Methodology; Software; Writing – original draft; Writing – review & editing. Mohammad Alkhawashki: Conceptualization; Investigation; Writing – original draft; Writing – review & editing. James Ross: Data curation; Investigation; Writing – original draft; Writing – review & editing. Jérôme Sallet: Conceptualization; Funding acquisition; Investigation; Resources; Writing – original draft; Writing – review & editing. Marcus Kaiser: Conceptualization; Funding acquisition; Investigation; Resources; Supervision; Writing – original draft; Writing – review & editing.

## FUNDING INFORMATION

Jérôme Sallet, Wellcome Trust (https://dx.doi.org/10.13039/100010269), Award ID: 203139/Z/16/Z. Jérôme Sallet, Sir Henry Dale Wellcome Trust Fellowship, Award ID: 105651/Z/14/Z. Jérôme Sallet, IDEXLYON (https://dx.doi.org/10.13039/501100024235), Award ID: IDEX/IMP/2020/14. Cyril Atkinson-Clement, Engineering and Physical Sciences Research Council (https://dx.doi.org/10.13039/501100000266), Award ID: EP/W004488/1 and EP/X01925X/1. James Ross, Engineering and Physical Sciences Research Council (https://dx.doi.org/10.13039/501100000266), Award ID: EP/W004488/1 and EP/X01925X/1. Marcus Kaiser, Engineering and Physical Sciences Research Council (https://dx.doi.org/10.13039/501100000266), Award ID: EP/W004488/1 and EP/X01925X/1. Marcus Kaiser, Guangci Professorship Program of Rui Jin Hospital, Award ID: Shanghai Jiao Tong University.

## Supplementary Material



## TECHNICAL TERMS

Transcranial ultrasound stimulation: A noninvasive neuromodulation technique that reaches deep brain areas and has high spatial resolution.
Redundancy: The repeated information we can obtain from any of the variables in the system.
Synergy: The extra information we get if we observe all the variables in the system together.
Structural connectivity: Anatomical links connecting two brain regions.
Modularity: A quantification of the network’s capability to be segregated into clusters.
Global efficiency: A quantification of the network communication efficiency.
