# Supplementary material for: Transcranial ultrasound stimulation effect in the redundant and synergistic networks consistent across macaques
Source: Netw Neurosci. 2024 Dec 10;8(4):1032–50. doi: 10.1162/netn_a_00388 (PMC11674579; doi:10.1162/netn_a_00388)
Supplement: Supplementary file 1 [file netn-8-4-1032-s001.pdf]

Journal: **NETWORK NEUROSCIENCE**

RESEARCH

Transcranial ultrasound stimulation effect in the redundant and synergistic networks consistent across macaques

Marilyn Gatica[1,2*], **Cyril Atkinson-Clement**[1], **Pedro A. M. Mediano**[3,4], **Mohammad Alkhawashki**[1], **James Ross**[1], **Jèrôme Sallet**[5,6], and **Marcus Kaiser**[1,7,8]

[1]Precision Imaging, School of Medicine, University of Nottingham, United Kingdom

[2]NPLab, Network Science Institute, Northeastern University London, London, United Kingdom

[3]Department of Computing, Imperial College London, London, United Kingdom

[4]Division of Psychology and Language Sciences, University College London, London, United Kingdom

[5]Wellcome Centre for Integrative Neuroimaging (WIN), Department of Experimental Psychology, University of Oxford, Oxford, United Kingdom

[6]Univ Lyon, Université Lyon 1, Inserm, Stem Cell and Brain Research Institute U1208, Bron, France

[7]School of Computing Science, Newcastle University, United Kingdom

[8]Rui Jin Hospital, Shanghai Jiao Tong University, Shanghai, China

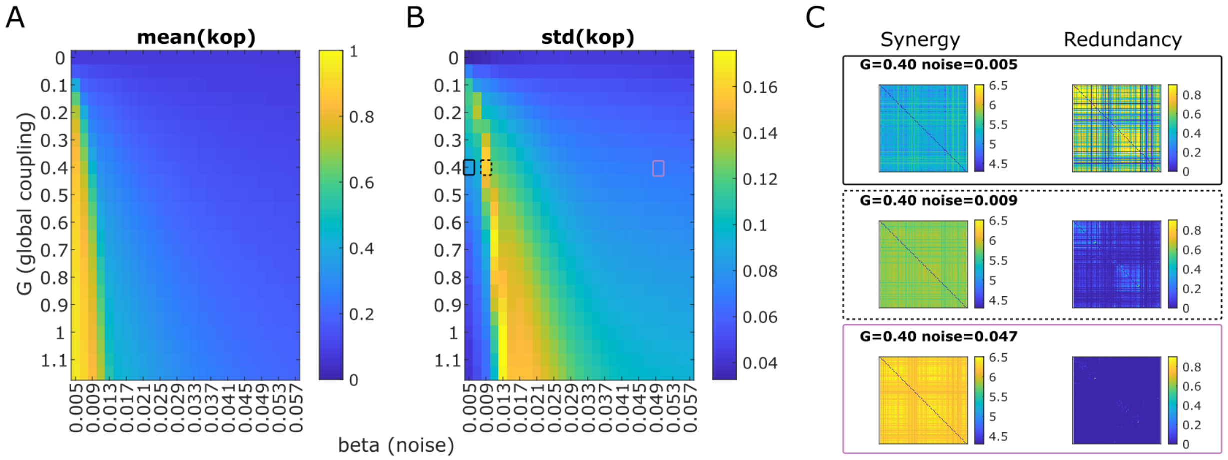

Figure 1. Hopf model simulations with N = 140 oscillators coupled with an average structural connectivity (across the three macaques), varying the parameters global coupling (G) and noise (β). **A:** The mean of the kuramoto order parameter (KOP) quantifying synchrony. **B:** The standard deviation of the KOP, as a measure of metastability. **C:** Redundant and synergistic matrices for $G = 0.4$ and three noise values.

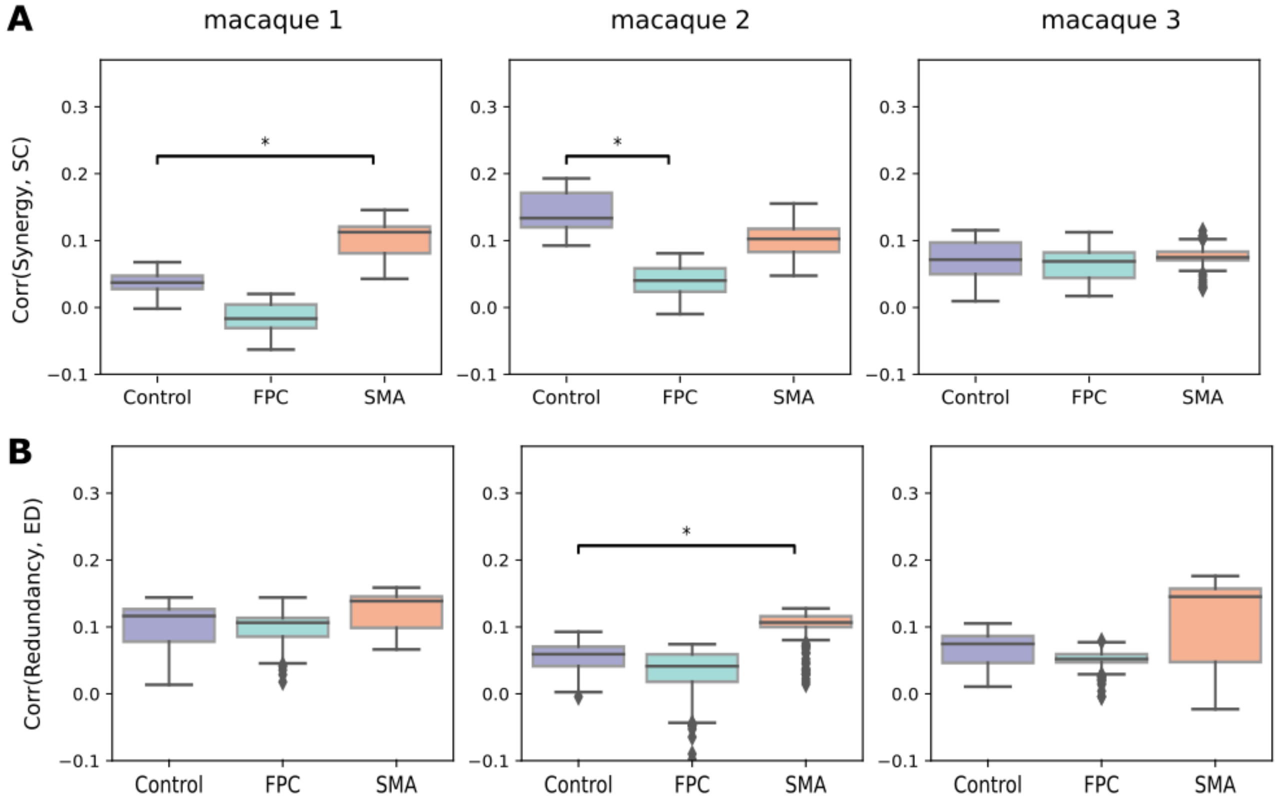

Figure 2. **A:** Correlation between the structural connectivity (SC) and the synergy. **B:** Correlation between the Euclidean distance (ED) and redundancy per experiment and macaque. The y-axis values contain the Spearman's rank correlation coefficient. The colors represent the control (non-TUS) and the two experiments: SMA-TUS and FPC-TUS. We corrected by Bonferroni and effect size bigger than 0.8.

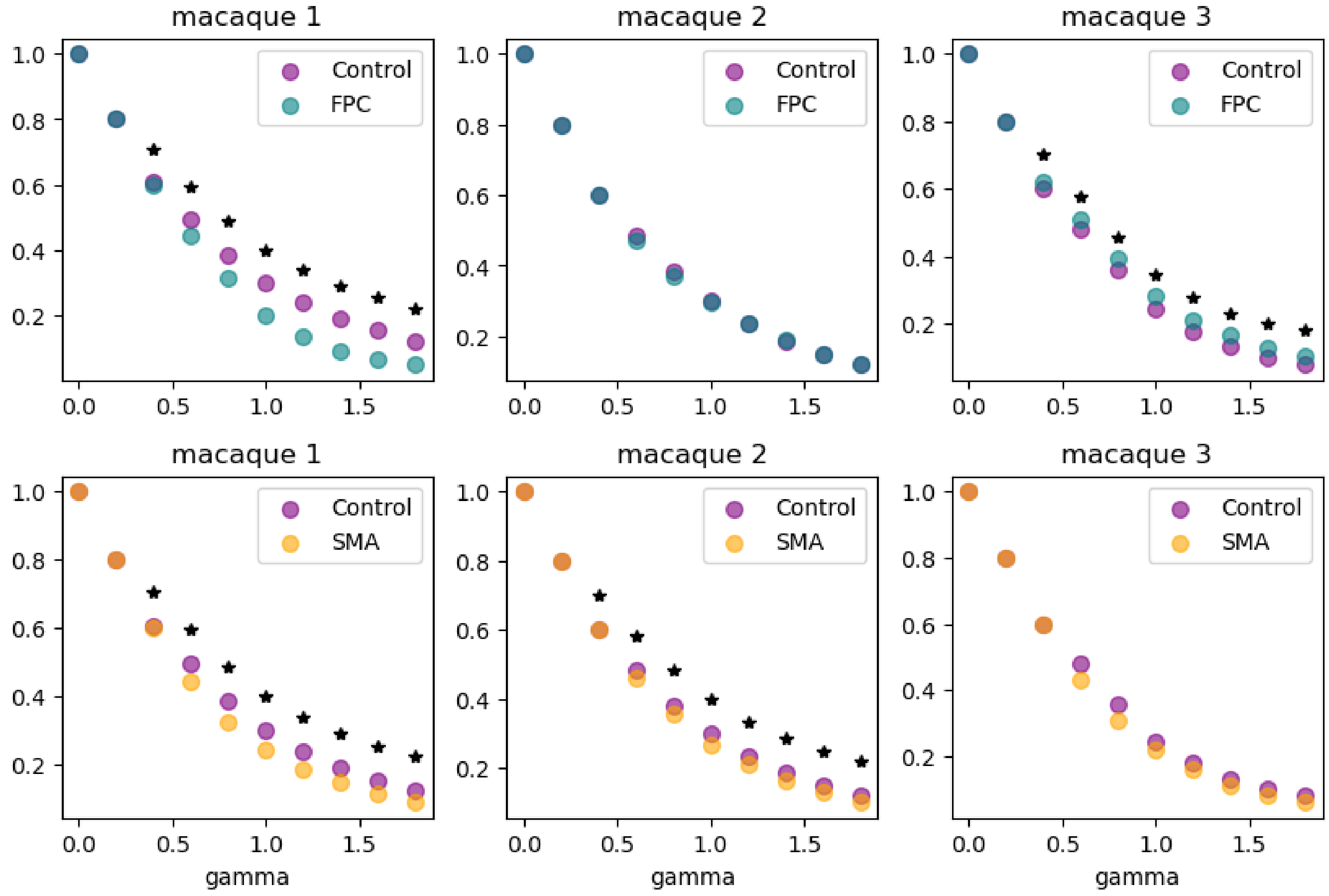

Figure 3. Median modularity value (segregation) of the redundancy matrix, per macaque and experiment. The modularity detection algorithm includes one free parameter, gamma, which controls the resolution of the clusters. Larger clusters are detected when gamma is between 0 and 1, while values greater than one result in smaller clusters. Per each parameter gamma, we computed the Wilcoxon rank-sum test to compare controls and the experiment (second row: SMA-TUS, first row: FPC-TUS). Te asterisk marks represent the significant differences (p-values corrected by Bonferroni and effect size bigger than 0.8).

Supplementary matherials and methods

We simulated the brain activity using a supercritical Hopf bifurcation model (Stuart-Landau oscillators)(Coronel-Oliveros et al., 2024; Deco et al., 2019). The following ordinary differential equations define the dynamic per each node i:

$$\frac{dx_i(t)}{dt} = a_i x_i(t) - \left[x_i^2(t) - y_i^2(t)\right] x_i(t) - w_i y_i(t) + G\sum_{j=1}^{n} M_{ij}\left(x_j(t) - x_i(t)\right) + \beta\eta_i(t)$$
$$\frac{dy_i(t)}{dt} = a_i y_i(t) - \left[x_i^2(t) - y_i^2(t)\right] y_i(t) - w_i x_i(t) + G\sum_{j=1}^{n} M_{ij}\left(y_j(t) - y_i(t)\right) + \beta\eta_i(t)$$

Where $y(t)$ corresponds to the imaginary component, and the real component of the time series, $x(t)$, simulated the BOLD-like signals. We set the oscillation frequency $f_i = 0.05$ Hz for overall nodes and the bifurcation parameter $a = 2$. The brain areas are coupled with the structural connectivity M (computed as the average structural connectivities across the three macaques). G represents the global coupling, and $\eta_i(t)$, with β the standard deviation, the external Gaussian noise. We ran 17 minute simulations with an integration step of 100ms in the Euler-Maruyama integration scheme. The simulated time series were band-pass filtered between 0.0025 and 0.05 Hz, as in the empirical data. We used the Python code to simulate the Hopf model, freely available at: `https://github.com/carlosmig/StarCraft-2-Modeling.git` (Coronel-Oliveros et al., 2024)

REFERENCES

Coronel-Oliveros, C., Medel, V., Orellana, S., Rodiño, J., Lehue, F., Cruzat, J., … Ibáñez, A. (2024). Gaming expertise induces meso-scale brain plasticity and efficiency mechanisms as revealed by whole-brain modeling gaming expertise, neuroplasticity and functional dynamics. *Neuroimage*. doi: 10.1016/j.neuroimage.2024.120633

Deco, G., Cruzat, J., Cabral, J., Tagliazucchi, E., Laufs, H., Logothetis, N. K., & Kringelbach, M. L. (2019). Awakening: Predicting external stimulation to force transitions between different brain states. *Proceedings of the National Academy of Sciences of the United States of America*. doi: 10.1073/pnas.1905534116
